# Examining the perceptions and behaviors of Gambian adults in response to COVID-19 social mitigation strategies

**DOI:** 10.11604/pamj.2020.37.238.25925

**Published:** 2020-11-16

**Authors:** Mat Lowe

**Affiliations:** 1 Society for the Study of Women´s Health, Old Yundum, The Gambia

**Keywords:** COVID-19, Gambia, coronavirus, perceptions, behaviors

## Abstract

The objective of this study was to examine the perceptions and behaviors of Gambian adults in response to COVID-19 social mitigation strategies. An online survey of 200 respondents was conducted. The survey inquired about respondents´ motivation to comply with a social distancing strategy and their ability to adopt 3 recommended social distancing strategies (avoiding public transport without wearing facemask, avoiding public gatherings and self-isolation). Respondents were also asked about the level of trust they had in the information about COVID-19 from the government and their confidence in the handling of the COVID-19 situation by the authorities. Fifty two percent (52%) of respondents reported that they would be motivated to comply with a social distancing strategy because they believed it is the right thing to do. Avoiding public transport without wearing facemask (n=154, 78.9%), followed by avoiding public gatherings (n=143, 73.3%) were considered to have high to very high capacity to adopt ratings among respondents. Whereas, only (n=132, 68.7%) thought that their ability to self-isolate, would be high to very high. Only (n=87, 44.2%) stated that they have high to very high level of trust in the information about COVID-19 from the government. The rest, (n=110, 55.8%) ranked their trust level as intermediate, low, very low or don´t know. Majority of respondents (n=114, 58.7%) disagreed to strongly disagreed that the authorities are doing a good job in handling the COVID-19 situation. These findings can be used to improve adoption of COVID-19 mitigation strategies and ensure trust and confidence in response efforts.

## Introduction

Since the first case of COVID-19 was confirmed in the Gambia on March 17^th^ 2020 [1], people and communities have been required, both voluntarily and mandatorily by law, to rapidly adoptvarious social mitigation strategies including self-isolation, keeping physical distances and avoiding public gatherings to prevent further spread of the virus. These social mitigation strategies were rarely used before, which means a large proportion of the population do not have prior experience undertaking these strategies [2]. This may also mean that people may find it difficult to comply with these strategies. The aim of this study was to examine the perceptions and behaviors of Gambian adults in response to COVID-19 social mitigation strategies.

## Methods

**Study design and setting:** a cross-sectional survey was used in this study. The survey was conducted among the adult population of the Gambia, from 7^th^ August 2020 to 30^st^ August 2020.

**Data collection:** given the social distancing measures and other restrictions, data were collected online, via a self-administered questionnaire, using Google Form. Respondents were asked to complete a survey link that was sent to them through instant message via WhatsApp application and Facebook. The instant message requested respondents to provide responses to the survey questions and forward the survey link to their contacts on WhatsApp for them to also participate in the survey and complete the survey questionnaire. The survey was composed of four main questions.

The first question asked respondents to comment on the factors that would motivate them to comply with a social distancing strategy. Response choices to this question include, a) I believe it is the right thing to do, b) to protect my community from infection, c) to protect myself from infection, d) to protect my family from infection, and e) all of the above.

The second question asked respondents to rate their ability to adopt 3 recommended social distancing strategies, a) avoiding public transport without wearing face masks, b) avoiding public gatherings like markets and restaurants, c) self-isolate in one room, if infected. Respondents were asked to rate their ability to carry out each of the 3 different strategies, with possible response choices ranging from a score of 1 for very high and 5 for very low.

The third question asked respondents to indicate the level of trust they have in the information about COVID-19 coming from the government. Response choices to this question ranged from a score of 1 for very high and 5 for very low. The fifth question asked respondents to rate how much they agree or disagree with the statement that, “In general, the authorities are doing a good job of dealing with the COVID-19 situation”. Responses choices to this statement were put on a five-point Likert scale ranging from 1 for strongly disagree to 5 strongly agree. The survey questions were adapted with permission from a published study in Australia [2].

The survey aimed to maximize reach and gather data from as many respondents as possible but ended up recruiting 200 respondents (18 years and older). The survey sample of 200 respondents (18 years and older) is representative of the adult population in the Gambia, which is divided into 3 age groups: a) 10-19 years; b) 20-39 years; and c) 40 years and above. According to the 2013 Population and Housing Census, the adult population of the Gambia that falls into each age group is estimated at 35% for 10-19; 42% for 20-39 and 23% for those aged 40 years and above.

**Data analysis:** for the data analysis, following submission of survey response from respondents, the summary of individual responses was presented in charts and was copied to clipboard and paste to an untitled Google Form. The individual responses were also exported and downloaded in excel file format and used to validate the data. Results were calculated and presented using simple frequency and percentage.

**Ethical considerations:** as this study represents an assessment of perceptions and behaviors towards COVID-19 social mitigation strategies and no data on medical conditions was collected, ethical approval was not sought. Yet, the study followed ethical principles guiding the conduct of research on human subjects. On the first page of the survey questionnaire, respondents were informed about the background and objective of the survey and their reserved rights to participate or not to participate, or to withdraw at any time without giving reason for their withdrawal. They were also assured of the confidentiality of the data and data collection. Respondents aged 18 years and older, understood the content of the questionnaire, agreed to participate in the survey and were instructed to complete the questionnaire. Consent, therefore, was implied if the respondent completed the survey and no personal identifiers were collected [2].

## Results

Overall, 200 respondents (18 years and older) participated in the online survey. Of these, 63.5% were male and 36.5% were female. The majority of respondents (66.3%) had attained university education as their highest qualifications.

**Figure 1:**
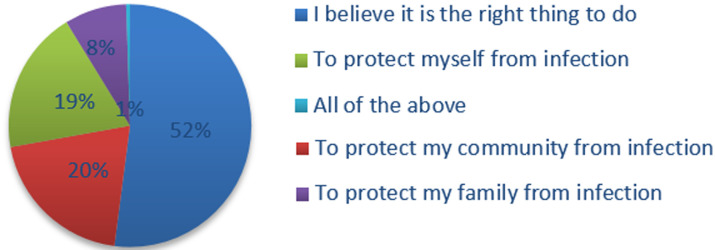
motivation to comply with a social distancing strategy

**Compliance and ability to adopt social mitigation strategies:** the survey found, 52% of respondents would comply with a social distancing strategy because they believed it is the right thing to do, followed by 20% and 19% of respondents who felt their compliance with a social distancing strategy would be to protect their communities and themselves from risk of infection (Figure 1). Avoiding public transport without wearing face mask (n=154, 78.9%), followed by avoiding public gatherings like markets and restaurants (n=143, 73.3%) were considered to have high to very high ability to carry out among respondents. Whereas, only (n=132, 68.7%) of respondents thought that their ability to self-isolate in one room, if infected would be high to very high.

**Trust and confidence in the information and handling of COVID-19 situation:** when asked about the level of trust they had in the information about COVID-19 from the government, only (n=87, 44.2%) stated they have high to very high level of trust, whereas the larger majority (n=110, 55.8%) ranked their trust level as intermediate, low, very low and others don´t know (Table 1). In addition, the survey found that, only (n=80, 41.2%) of respondents agreed to strongly agreed that the authorities are doing a good job in dealing with the COVID-19 situation, whereas the rest (n=114, 58.7%) disagreed to strongly disagreed (Table 1).

**Table 1:** level of trust in the information related to COVID-19 and perceptions towards response measures to COVID-19 from government

Question	Response (%)
What level of trust do you have in the information about COVID-19 from the Government?	
Very high	39 (19.8)
High	48 (24.4)
Intermediate	55 (27.9)
Low	34 (17.3)
Very low	20 (10.2)
Don´t know	1 (0.5)
**Statement**	**Response (%)**
In general, I think the authorities are doing a good job of dealing with the Covid-19 situation	
Agree	55 (28.3)
Strongly agree	25 (12.8)
Disagree	85 (43.8)
Strongly disagree	29 (14.9)

**Note:** % represents respondents who provided responses

## Discussion

This study suggests that the motivation to comply with a social distancing strategy among Gambian adults may be largely influenced by a sense of moral ideal, which also suggests that communication messages that are framed around a social collective action may be more effective response towards COVID-19, as argued by Seale H *et al*. [2]. The study also revealed that respondents considered their ability to avoid public transport without wearing face mask and avoid public gatherings like markets and restaurants to be slightly higher than their ability to self-isolate in one room, if infected. This finding is different from what was found by a study in Australia [2], which revealed that the majority of respondents agreed that they could self-isolate if necessary. It is not surprising that the ability to self-isolate has scored lower than the other strategies because in the Gambia most people live in extended families and usually share accommodations. This is a confirmation that self-isolation as a social distancing strategy may be difficult to carry out in the Gambia.

The study also showed that the majority of survey respondents ranked their level of trust in the information about COVID-19 coming from the government as either intermediate, low, very low, and others don´t know. Also, a little more than half of the respondents disagreed to strongly disagree with the statement that the authorities are doing a good job of dealing with the COVID-19 situation. What these findings suggest is that there is low level of trust and confidence on response efforts by the government and authorities, which must be considered. Experience from the 2014 Ebola epidemic in West Africa showed that mistrust about the response strategies led to poor acceptance among people, which resulted in escalation of the epidemic and contributed to the high mortality recorded in Guinea, Liberia and Sierra Leone [3, 4].

Although this study has provided insight into our understanding of the perceptions and behaviors of Gambian adults in response to COVID-19 social mitigation strategies, the findings are subject to at least four limitations, which must be considered when interpreting the findings. First, responses were self-reported and could be subject to social desirability bias. Second, while survey respondents included people of different ages, they may still be different from the general Gambian population on a variety of characteristics. Third, the study was limited to only those with internet access, which may also bias the findings. Lastly, it is possible that respondents looked up the answers to some of the questions online prior to answering, which may have biased the findings from factual rather than opinion-focused [5]. Despite these limitations, the findings have important implications for improving adoption of recommended COVID-19 social mitigation strategies and in ensuring trust and confidence in national response efforts.

## Conclusion

The results of this study can be used by the government and other relevant stakeholders to improve adoption of recommended COVID-19 social mitigation strategies and to ensure trust and confidence in national COVID-19 response efforts.

### What is known about this topic

- The outbreak of COVID-19 has led to the adoption of various social mitigation strategies;
- The social mitigation strategies adopted were rarely used before.

### What this study adds

- Gambian adults are highly motivated by a sense of moral ideal to adopt a social distancing strategy;
- Their ability to adopt recommended COVID-19 social mitigation strategies, such as facemask wearing and avoidance of public gatherings is reportedly high;
- The Gambian adults have limited trust in the information about COVID-19 coming from the government and their confidence towards the handling of the COVID-19 situation by the authorities is also limited.
